# Dominance determines fish community biomass in a temperate seagrass ecosystem

**DOI:** 10.1002/ece3.7854

**Published:** 2021-07-06

**Authors:** Aaron M. Eger, Rebecca J. Best, Julia K. Baum

**Affiliations:** ^1^ Department of Biology University of Victoria Victoria BC Canada; ^2^ School of Earth and Sustainability Northern Arizona University Flagstaff AZ USA; ^3^ Present address: School of Biological, Earth, and Environmental Sciences University of New South Wales Sydney NSW Australia

**Keywords:** biodiversity, community ecology, ecosystem function, eelgrass, functional diversity

## Abstract

Biodiversity and ecosystem function are often correlated, but there are multiple hypotheses about the mechanisms underlying this relationship. Ecosystem functions such as primary or secondary production may be maximized by species richness, evenness in species abundances, or the presence or dominance of species with certain traits. Here, we combine surveys of natural fish communities (conducted in July and August 2016) with morphological trait data to examine relationships between biodiversity and ecosystem function (quantified as fish community biomass) across 14 subtidal eelgrass meadows in the Northeast Pacific (54°N, 130°W). We employ both taxonomic and functional trait measures of diversity to investigate whether ecosystem function is best predicted by species diversity (complementarity hypothesis) or by the presence or dominance of species with particular trait values (selection or dominance hypotheses). After controlling for environmental variation, we find that fish community biomass is maximized when taxonomic richness and functional evenness are low, and in communities dominated by species with particular trait values, specifically those associated with benthic habitats and prey capture. While previous work on fish communities has found that species richness is often positively correlated with ecosystem function, our results instead highlight the capacity for regionally prevalent and locally dominant species to drive ecosystem function in moderately diverse communities. We discuss these alternate links between community composition and ecosystem function and consider their divergent implications for ecosystem valuation and conservation prioritization.

## Graphical abstract

We tested whether more fish species living in seagrass meadows in northern Canada was related to the function of that ecosystem. Instead of more species equally more functioning, we found that dominant species were the most important factor.

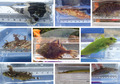

## 1 INTRODUCTION

Understanding the mechanisms underlying biodiversity–ecosystem function (BEF) relationships is critical both for developing predictive ecological theory and for effective ecosystem management. Each hypothesis has a distinct implication about the “value” of a species to an ecosystem. For instance, high ecosystem functioning can result from the distinct actions of all species present in a diverse community. Under this “complementarity hypothesis” (Tilman et al., 2001), a greater number of species with distinct traits will be able to use a greater range of resources and perform a greater range of functions (Davies et al., 2011; Jenkins, 2015). Thus, the loss of any single species would negatively impact ecosystem function. In contrast, other hypotheses suggest that a few species contribute disproportionately to ecosystem function due to their large population size or ecological impacts. For example, under the “selection hypothesis”, higher species richness increases ecosystem function only because it increases the chance that a community will include one of these high‐impact species, with additional species contributing only marginal gains to ecosystem function (Loreau & Hector, 2001). High‐impact species may also contribute disproportionately due to their traits and also their relative abundance: The “biomass ratio” or “dominance” hypothesis (Grime, 1998) predicts that ecosystem function will increase with the relative abundance or dominance of those particularly important species (Finegan et al., 2015; Zhu et al., 2016). If ecosystem management seeks to maximize function, the implication of either of these latter hypotheses is that the identification and protection of specific species are more important than maintaining the greatest number of species. Finally, the effects of species diversity may be outweighed by direct effects of abiotic factors on ecosystem functioning (Huston & McBride, 2002), and management actions would need to focus on mitigating stressors like temperature or pollution (Srivastava & Vellend, 2005) or selecting areas with preferable abiotic conditions (Keppel et al., 2012). Because of their distinct implication for management, quantifying the relative importance of these proposed mechanisms is important for accurately assessing losses of ecosystem function, prioritizing future conservation efforts (Cardinale et al., 2012; Steffen et al., 2007), and planning restoration efforts that achieve ecosystem function goals (Laughlin, 2014).

One way to improve our ability to identify mechanisms linking species diversity and ecosystem function is to assess both organismal traits and taxonomic metrics in BEF analyses. Although taxonomic metrics can be proxies for the niche space occupied by community members, they do not directly quantify niche diversity or overlap, a central element of BEF hypotheses (Fargione et al., 2007; Hooper et al., 2005). In contrast, functional (i.e., trait‐based) metrics such as functional richness (the size of trait space occupied by a community), functional evenness (how abundance is distributed among trait values), and functional dispersion (the dissimilarity of trait values) directly incorporate niche overlap among species (Laliberté & Legendre, 2010; McPherson et al., 2018; Villéger et al., 2008). In addition, examining the community weighted mean (CWM) values of individual traits (Swenson, 2014) enables inference about how EF is related to particular trait values.

The use of both functional and taxonomic metrics to differentiate between BEF mechanisms has been widely applied in terrestrial systems (particularly plant communities), but less is known about these relationships in marine ecosystems. Past BEF work in terrestrial systems has found contrasting results, with some studies’ findings supporting the selection hypothesis (Wasof et al., 2018), others supporting complementarity (Duffy et al., 2017), and still others supporting abiotic factors as the main drivers of ecosystem function (Van Eekeren et al., 2010). BEF work in coastal marine systems has been limited (only 13 publications in van der Plas', 2019 review), and the evidence is dominated by one study with thousands of data points (Duffy et al., 2016). These results and others (Benkwitt et al., 2020; Danovaro et al., 2008; Mora et al., 2011) currently suggest that complementarity is the most common driver of ecosystem function in marine ecosystems. Further, in both terrestrial and marine systems, there is also recognition that more work is needed to assess BEF relationships in natural ecological communities. Although observational studies cannot independently manipulate different aspects of community diversity, they have the advantage of including real constraints on species assemblages across gradients of abiotic variation (Bannar‐Martin et al., 2018). Thus, they provide an important source of complementary evidence that is necessary for understanding BEF relationships in the field.

Globally, coastal seagrass systems provide many important ecosystem functions and services that are often linked to biodiversity at multiple trophic levels. In addition to water filtration, carbon capture, and sediment stabilization (Cullen‐Unsworth & Unsworth, 2013; Larkum et al., 2006; Röhr et al., 2018), these habitats provide important fish and invertebrate nursery habitats (McDevitt‐Irwin et al., 2016) and directly or indirectly support additional levels of algal, invertebrate, fish, and bird biodiversity (Gilby et al., 2018; Phillips, 1984). Fishes are key components of seagrass ecosystems, which can directly promote healthy habitats by deterring herbivores (Eger & Baum, 2020) or indirectly promote grazers of epiphytic algae colonizing seagrass blades (Moksnes et al., 2008); their secondary production also feeds higher trophic levels that support commercial fisheries (Unsworth et al., 2019). Despite their global distribution and contribution to fisheries, few studies have examined BEF mechanisms in seagrass ecosystems (Allgeier et al., 2015; Duffy et al., 2015), particularly for fish communities. Thus, while there is evidence that genetic diversity within some seagrasses can promote productivity and stability (Abbott et al., 2017; Hughes & Stachowicz, 2004), as can species diversity in invertebrates, such as snails and crustaceans (Duffy et al., 2015), we know much less about the mechanisms driving secondary production in the diverse fish communities found in seagrasses.

In this study, we address these gaps in understanding for a critical marine ecosystem by testing the importance of contrasting BEF mechanisms of fish communities in temperate eelgrass (genus: *Zostera*) ecosystems. We achieve this goal by considering both functional and taxonomic diversity metrics and using the shape and direction of BEF relationships (Table 1) to identify the underlying mechanisms in communities that reflect natural variation in fish species composition and environmental conditions. If ecosystem function (EF) is related to the complementarity effect, we expect to see positive, nonsaturating relationships between EF and biodiversity, whether it is measured as richness (Mora et al., 2014), taxonomic diversity (Cavanaugh et al., 2014), or through measures of morphological traits such as functional dispersion (Gagic et al., 2015) or functional evenness (Mason et al., 2005; Table 1). If EF is instead only related to the presence of particular species, we expect positive but saturating relationships between EF and functional and taxonomic richness, taxonomic diversity, and functional dispersion (Mora et al., 2014), and no relationship between EF and evenness (Table 1). If it is not just presence but dominance (i.e., high relative abundance of certain species) that drives EF, then high diversity and evenness should instead be associated with decreased function due to lower contributions from those particularly important species (Table 1). In this case, we expect negative relationships between EF and functional evenness (Grime, 1998), taxonomic metrics, and functional dispersion, and strong relationships with CWM trait values (Finegan et al., 2015; Zhu et al., 2016). Lastly, if EF is best explained by abiotic or habitat factors, we expect to find null relationships between EF and the diversity metrics and strong relationships between EF and the environmental metrics.

**TABLE 1:** Predicted relationships between biodiversity (B) metrics and ecosystem function (EF) under each BEF hypothesis

Predictor variable	Complementarity	Selection	
		Presence	Dominance (mass ratio)
Taxonomic richness	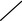	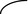	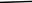
Taxonomic diversity	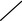	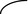	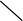
Functional richness	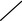	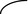	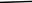
Functional dispersion	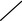	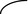	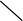
Functional evenness (multi‐ or one‐dimensional)	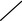	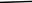	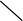
CWM traits	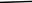	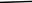	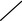 or 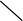

## 2 MATERIALS AND METHODS

### 2.1 Fish community surveys

**FIGURE 1:**
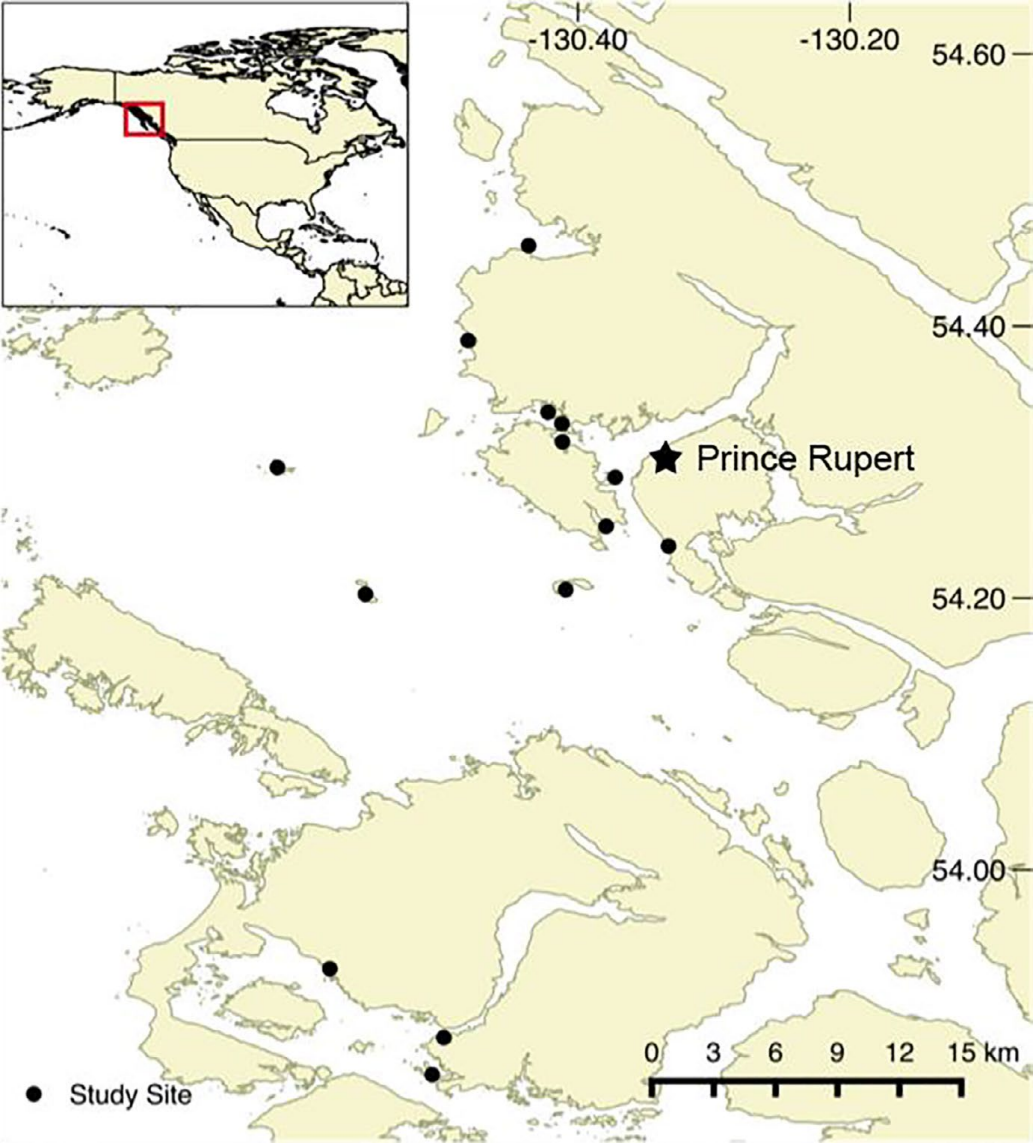
Study area with surveyed sites on the northern coast of British Columbia, Canada. Source: Canadian Hydrographic Service

In the summer of 2016, we surveyed fish communities in fourteen subtidal eelgrass meadows on Canada's west coast in northern British Columbia (Figure 1). To minimize seasonal variability, we completed all surveys within a five‐week period (1 July to 5 August). To access the subtidal portion of each meadow, we surveyed at the day's low‐low tide and only on days with a tidal height of less than 1 meter depth as measured by chart datum at mean low‐low water. Surveying consisted of duplicate beach seines (Guest et al., 2003) using a seine that measured 10 m in length, 3 m in depth at the center, tapered to 1 meter depth at each end, and with a mesh size of 6 mm as measured along the diagonal. The area sampled was approximately 60 m^2^ per site. After each seine haul, we collected the fish in tubs on shore. To avoid recounting released individuals, we conducted both hauls before identification. Once both sets were completed, we counted all individuals and measured the fork length (to the nearest millimeter) of the first 20 individuals of each species at each site. This method is the standard protocol for surveying nearshore waters, but as with most sampling methods, it misses some individuals (i.e., here, fishes present only at high tide or in deeper waters). As a result, our results are specific to the well‐sampled subset of the community. We quantified site‐level eelgrass density at 11 sites by collecting all shoots within nine haphazardly thrown 25 × 25 cm quadrats at a site and counting them in the laboratory. We also quantified temperature and pH at each site using a Hanna HI^®^ meter and extracted site‐level salinity from a GIS layer at a 500 × 500 m spatial resolution (Foreman et al., 2008).

### 2.2 Functional trait measures

**FIGURE 2:**
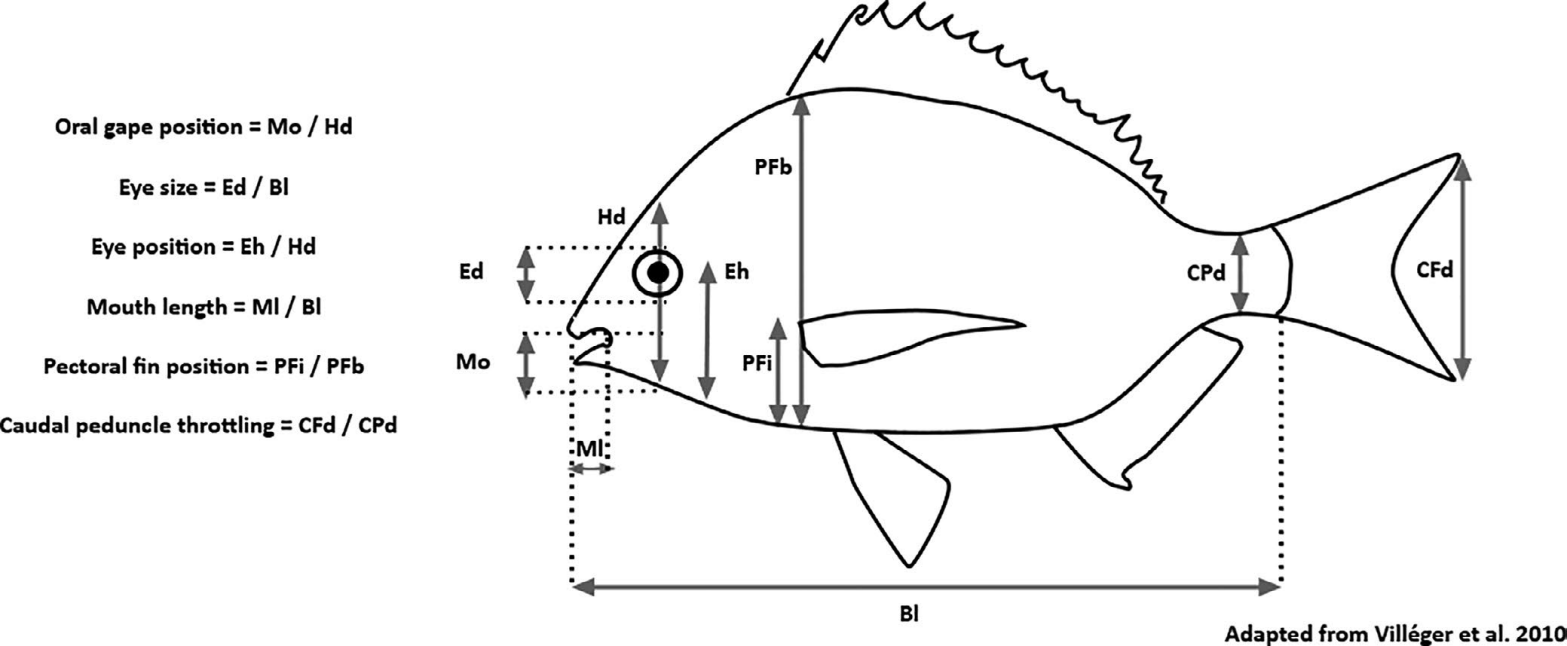
Morphometric traits measured from photographs of individuals of each of the 34 species. All traits (except for body length) are expressed relative to other body parts (total head, body size, or caudal peduncle depth) and are therefore independent of variation in total size. Trait abbreviations are as follows: Bl, body length; CFd, caudal fin depth; Cpd, caudal peduncle depth; Ed, eye depth; Eh, eye height; Hd, head depth; Ml, mouth length; Mo, mouth opening; PFb, body depth at pectoral fin; PFi, height of pectoral fin

We calculated seven species‐level functional traits: mouth length, oral gape position, eye size, eye position, pectoral fin position, caudal peduncle throttling, and body length (Figure 2). We chose these specific traits because of their connection to resource acquisition, either through size, visual acuity, mobility, or position in the water column, which should combine to determine each species’ niche within the community (Villéger et al., 2010). To obtain these morphometric trait measures, we photographed representative individuals of each species (although not every species was photographed at every site) and used the image processing software ImageJ (Rasband, 1997) to make the measurements (Villéger et al., 2010). Each trait (except for body length) is size‐standardized and thus constrained between zero and one. Species‐level functional trait values were obtained by taking the mean value of each trait across all individuals measured for each species (372 total measures, mean per species = 9.57, standard deviation = 11.91). One species, *Cymatogaster aggregata*, was an exception because it was found in both juvenile and adult stages. As a result, a single mean would mask bimodal variation in traits, and we therefore considered these two stages as functionally distinct and calculated separate morphological metrics for juveniles and adults. Other species were relatively similar in body size and were considered together. Photographs and trait measures were unobtainable for five of the rarest species (*Myoxocephalus polyacanthocephalus* (*N* = 3), *Rimicola muscarum* (*N* = 1), *Ascelichthys rhodorus* (*N* = 1), *Sebastes miniatus* (*N* = 1), and *Rhacochilus vacca* (*N* = 1), so they were dropped from the taxonomic and functional analyses.

### 2.3 Functional diversity

We quantified three metrics of multivariate functional diversity (functional richness, functional evenness, and functional dispersion) using the “FD” package (Laliberté & Legendre, 2010) in R. Calculation of each of these multivariate metrics first involved performing a principal coordinates analysis (PCoA) which reduces the complexity of multiple traits into two‐dimensional space. Functional richness is the volume of the polygon that connects the exterior points of this transformation (Villéger et al., 2008). Functional evenness, which is independent of functional richness, is a measure of how regularly traits are distributed throughout the 2‐D trait space and how abundance is distributed across those traits (Mason et al., 2005). Functional dispersion is calculated as the average distance to the abundance weighted centroid with the traits represented in n‐dimensional space (Laliberté & Legendre, 2010). The calculations for evenness and dispersion were done using the abundance weighted Gower's dissimilarity measure and included all the traits. The calculation for functional richness was not weighted by abundance and included six of the seven traits; caudal peduncle throttling was dropped from this calculation because it was found to be redundant in the PCoA. The resulting quality of the reduced space representation, which is analogous to *R* ^2^, was .93. Prior to conducting the PCoA, we standardized the morphometric traits to a mean of zero and standard deviation of one and gave all traits equal weighting.

To better understand which individual traits underlay the relationships between biomass and the multivariate functional diversity metrics, we also calculated two univariate metrics. We focused on understanding the evenness (and conversely the dominance) of individual traits because functional evenness was the only functional diversity metric to show a strong relationship with ecosystem function. First, we calculated univariate indices of functional evenness (i.e., functional regularity (Mouillot et al., 2005). Second, because we also wanted to understand the directional influence of specific trait values on community biomass, we calculated the community weighted mean (CWM, Swenson, 2014) of each trait. The CWM is calculated by taking the average trait value of the individuals at each site (e.g., S_1_ = 10 cm, S_2_ = 15 cm, S_3_ = 20 cm, CWM = 15 cm).

### 2.4 Taxonomic metrics

We used two taxonomic metrics, Chao's species richness and Chao's Shannon's diversity (hereafter “taxonomic diversity”). Both metrics are rarefied measures of taxonomic diversity, which use sample‐based rarefaction that incorporate abundance data. We rarefied our diversity metrics to account for the nonrandom distribution of fishes in the water column and for differences in species’ abundance at each site (Colwell et al., 2012; Gotelli & Colwell, 2011). The final values reported are the asymptotic estimates of diversity at each site. The calculations were done using the “iNEXT” function in the “iNEXT” (Hsieh et al., 2016) package in R.

### 2.5 Ecosystem function

Following Mora et al. (2011) and Duffy et al. (2016), we used community standing stock biomass (hereafter “community biomass”) as our measure of ecosystem function. Though this is not a rate, fisheries landings are often viewed as a type of ecosystem function or service (Holmlund & Hammer, 1999) and the biomass of an individual frequently predicts its contribution to a range of other ecosystem functions such as suspension feeding, nutrient uptake, and gross productivity (Davies et al., 2011). We estimated each individual's mass using its recorded length and published length–weight relationships, most of which were specific to fishes in eelgrass meadows in British Columbia (Siegle et al., 2014), while data for five species were only available from Fishbase.org (Froese & Pauly, 2010). Lengths were measured for the first 20 individuals of each species at each site. As such, lengths were not available for all individuals, so truncated density distributions based on maximum and minimum observed fish lengths were created using the “fitdistrplus” package in R (Delignette‐Muller & Dutang, 2015). The unmeasured individual lengths were then randomly sampled from these distributions. Lastly, biomass was summed at each site and logged to normalize its variance.

### 2.6 Statistical analyses

To examine the relationships between fish community biomass and each taxonomic and functional metric, we fitted generalized linear models (GLM) with a gamma distribution. We chose a gamma distribution because the data were non‐normal and contained positive nonzero values. We examined the univariate relationships between each of four environmental variables (temperature, salinity, pH, eelgrass density) and fish community biomass, but did not include these in our GLMs to avoid overparameterizing our model (i.e., by including multiple correlated fixed effects with a low sample size; Appendix S1). Instead, we included the geographic location (longitude) of the survey sites as a second fixed effect in each GLM. Because longitude was strongly correlated with the abiotic variables (Appendix S1), with sites farther from the coast tending to have lower temperature, higher salinity, higher eelgrass density, and less human disturbance, we considered it a proxy for the environmental relationships with fish biomass. We also tested for spatial autocorrelation in the biotic and abiotic variables among the surveyed sites using the Durbin–Watson test, but found no significant effects of spatial autocorrelation. The models were then evaluated for relative fit using the proportion of the deviance explained (*D^2^*), which is measured in comparison with a null model that includes only the intercept. This measure of fit was calculated using the R package “modEvA” (Barbosa et al., 2016). Predictors with the highest *D* ^2^ values were interpreted to be most important for explaining community biomass. As a secondary approach, we also assessed variables for statistical significance based on their *p* value (*p* < .05) and adjusted for multiple comparisons using the Holm–Bonferroni method and the *p.adjust* function in R (Holm, 1979), herein referred to as *p*_adj_ (*p* adjusted). All other statistical operations were also performed using the R programming language (R Core Development Team V 4.0, 2020). All data and associated code for graphing and analysis are archived in a GitHub repository and on Open Science Framework (https://github.com/baumlab, https://doi.org/10.17605/OSF.IO/6SQRF).

## 3 RESULTS

Species richness and community biomass varied substantially across study sites. Unrarefied richness ranged from 7 to 23 species per site, with a regional pool of 34 species. Of these 34 species, over three‐quarters (*N* = 26) were found at more than one site within the region. Community biomass ranged 10‐fold across sites, from 1,216 to 12,774 g (log_10_, 3.08 to 4.10) per site (approximately 60 m^2^). All sites were dominated (>50% of abundance) by three or fewer species (Appendix S2), and only 16 of the 34 species had median site biomass proportion values above 0.01 (Appendix S3). Of these 16 species, the two with the highest median proportion of site biomass were the surfperch, *Cymatogaster aggregata* (0.34), and the sculpin, *Leptocottus armatus* (0.21, Appendix S3).

### 3.1 Biodiversity–ecosystem function

**FIGURE 3:**
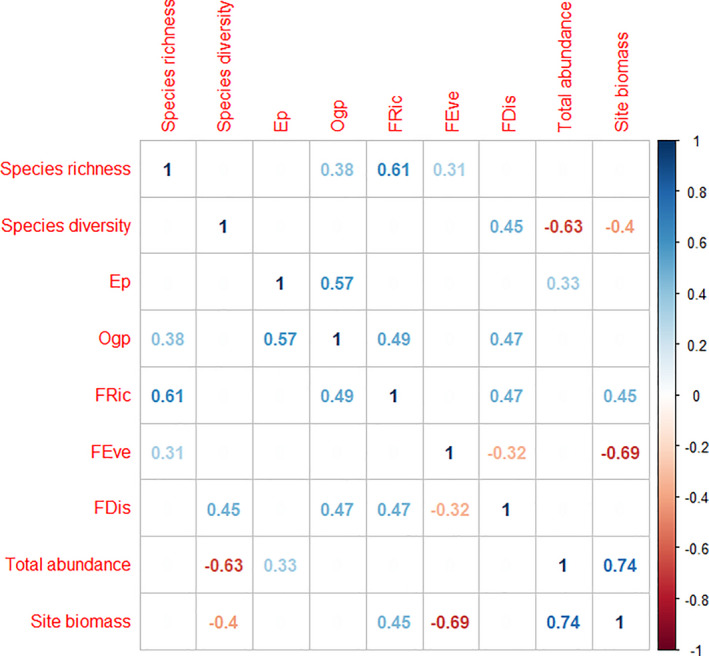
Correlations between diversity metrics, site abundance, and site biomass

**FIGURE 4:**
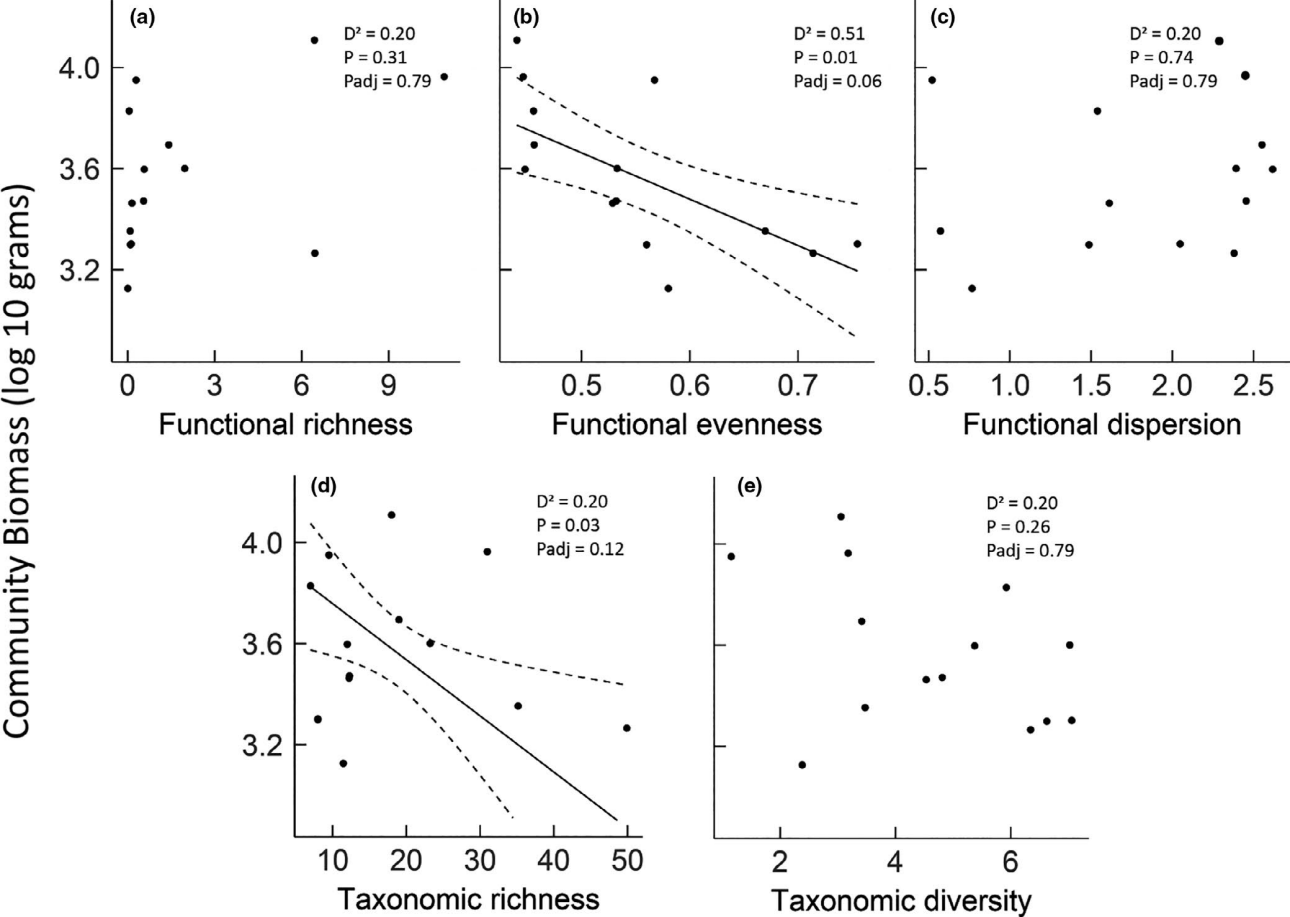
Relationships between the log site‐level fish community biomass and (a) functional richness, (b) functional evenness, (c) functional dispersion, (d) taxonomic richness, and (e) taxonomic diversity. The solid line (shown only for the significant relationships pre‐adjustment, *p*_adj_) is the regression line, and the dashed lines are twice the standard error

Fish community biomass was negatively related to both functional evenness (Figure 3), the biodiversity metric that explained the most deviance (*D* ^2^ = 0.51, Figure 4, *p* = .01, *p*_adj_ = .06), and to species richness (*D* ^2^ = 0.43, Figures 3 and 4, *p* = .03, *p*_adj_ = .12). The remaining biodiversity metrics, taxonomic diversity, functional richness, and functional dispersion did not explain high amounts of deviance (*D* ^2^ < 0.22, Figure 4). Additionally, none of the four environmental variables explained more than 0.2 of variance (Appendix S4). When included as a covariate, longitude (our environmental proxy) was never a significant predictor.

**FIGURE 5:**
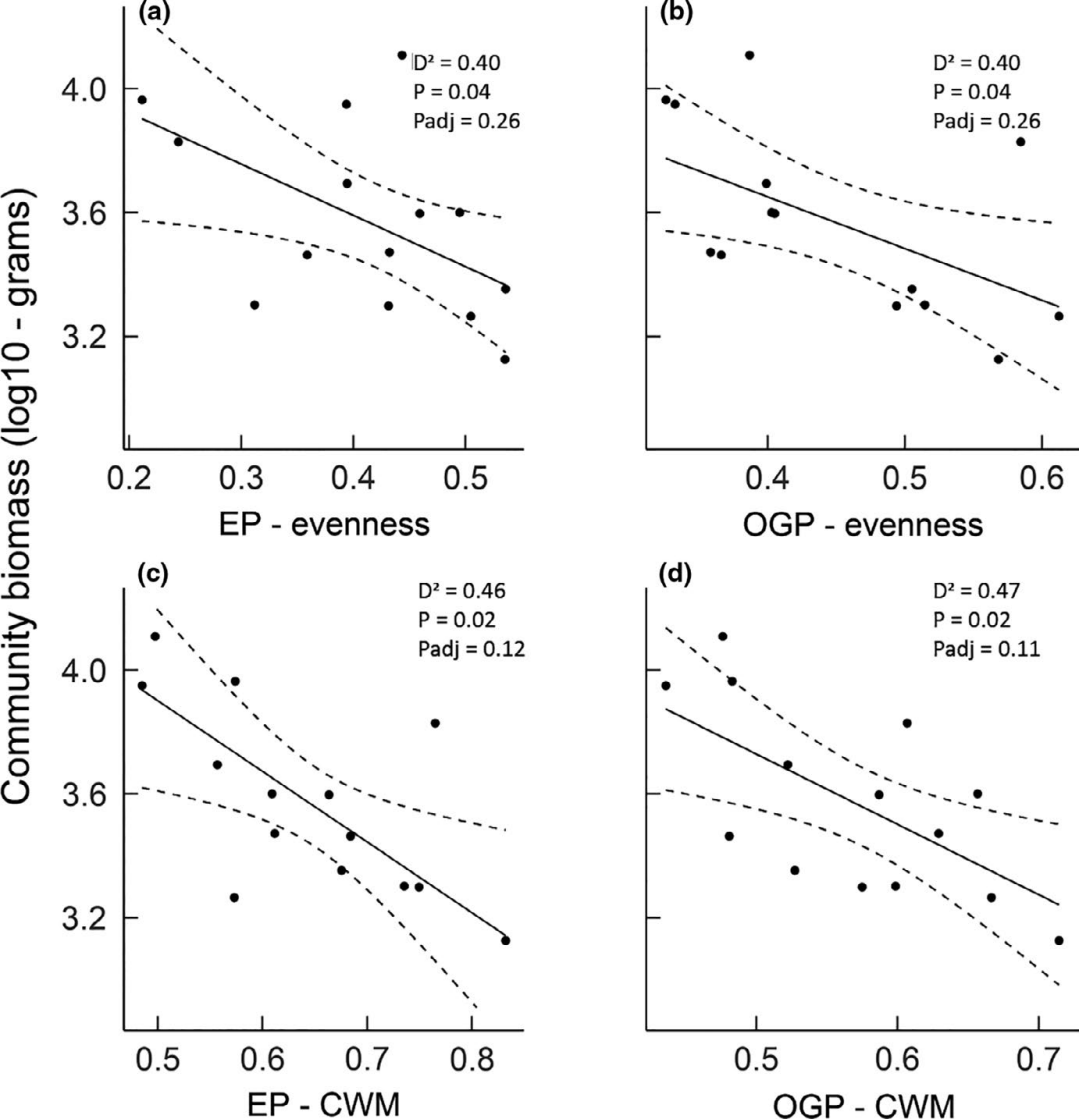
Relationships between the log site‐level community biomass and (a) evenness of eye position (EP), (b) evenness of oral gape position (OGP), (c) community weighted mean (CWM) of CWM of eye position, and (d) CWM of oral gape position. The solid line (shown only for the significant relationships pre‐adjustment, *p*_adj_) is the regression line, and the dashed lines are twice the standard error

The relationship between functional evenness and fish community biomass appeared to be associated with morphological trait values. Of the seven evenness trait values considered individually, two, lower evenness of eye position and oral gape position, explained high amounts of variance community biomass (*D* ^2^ = 0.40 for both vs. next largest *D* ^2^ = 0.21, Figure 5). The negative relationship between evenness and biomass indicates that community biomass was maximized in communities dominated by certain values of these traits. Similarly, the community weighted means of these two traits were the CWM values best able to predict fish community biomass. The CWM for eye position and oral gape position were both negatively related to community biomass (*D* ^2^ = 0.46 and 0.47, next largest *D* ^2^ = 0.25, Figure 5). The relationship between the average CWM length and community biomass at our sites was weaker, suggesting that gape and eye traits are more important than individual size (*D* ^2^ = 0.34).

## 4 DISCUSSION

We found clear evidence that, in our system, high standing stock biomass of fish in eelgrass meadows was best explained by the dominance of species with particular trait values rather than complementary resource use of a diverse species assemblage. Sites with low species diversity and high dominance of species with certain traits had the highest ecosystem function (total fish community biomass) of eelgrass‐associated fish communities. This conclusion is supported by five out of six of the relationships predicted in Table 1; specifically, community biomass decreased with increasing taxonomic richness and functional evenness (both the multivariate and the two univariate measures) and was strongly related to two CWM trait measures. Community biomass was also not well predicted by the environmental factors. These results are contrary to similar studies of fishes in coastal marine environments (Benkwitt et al., 2020; Duffy et al., 2016; Mora et al., 2011), which found positive relationships between community biomass and taxonomic and functional diversity metrics, and a strong influence from environmental variables, albeit over larger gradients.

### 4.1 Dominance vs. diversity as drivers of ecosystem function

Communities with fewer species had the highest levels of ecosystem function among our study sites (Figure 3). In fact, the abundance and distribution of dominant species played a role at two spatial scales. First, at the level of an individual community (i.e., a site), communities with the highest function had the highest local dominance of high‐contributing species. Second, the two most dominant species (*Cymatogaster aggregata* and *Leptocottus armatus*) had a high degree of regional occupancy (Appendix S3) such that greater species richness at the community level corresponded to substituting individuals of these dominant species for individuals of rare species with overall low biomass that contributed less to ecosystem function. This mechanism helps explain why we failed to find a positive relationship between function and taxonomic richness, as predicted under the complementary hypothesis. Our results suggest that many species contribute very little to certain components of ecosystem function and increased species richness correlates with fewer dominant individuals.

Our main result, namely that low levels of species richness and evenness best predict high biomass in temperate eelgrass fish communities, is contrary to past research suggesting that more coastal fish species equates to more biomass (Duffy et al., 2016; Mora et al., 2011). For example, in an extensive analysis of 1,844 sites on shallow hard substrate, Duffy et al. (2016) found an overall positive effect of species richness on community biomass; more so, they found this effect was strongest in temperate ecosystems such as ours. Though they studied rocky reefs and we studied a seagrass on soft substrate, we do not believe this distinction is enough to explain our contrasting results. Instead, we suggest two differences between the two studies that may explain the different conclusions. First, the range in species richness was quite different; Duffy et al.'s (2016) surveys in temperate regions ranged from ~1 to 10 species whereas our surveys ranged from 7 to 23 species. Therefore, Duffy et al.'s (2016) results might have arisen because their communities had very few species, in which the addition of even a single species resulted in a substantial fulfillment of niche space (Stuart‐Smith et al., 2013). This result could occur if species that are important contributors to community biomass are not regionally well distributed and are not reliably found in almost all communities at minimum species richness levels. Because our study contained moderately species‐rich ecosystems, the species count may have been past the saturation point on the richness–function curve that is often seen in BEF relationships (Morris et al., 2014). Second, we used beach seines to survey, which are more likely to detect rare, singleton species (Baker et al., 2016) than the visual surveys used in Duffy et al. (2016). These rare, singleton species would have contributed very little biomass, but still increased the species count, and thus possibly prevented a relationship between species richness and community biomass. Using similar sampling techniques (nets rather than visual surveys), Maureaud et al. (2019) also found that dominance was the best predictor of fish biomass in communities with species richness values similar to ours (7–44 species). Lastly, our results are representative of eelgrass ecosystems in the Pacific Northwest (Robinson & Yakimishyn, 2013) and were biogeographically similar to each other. Other temperate marine ecosystems with lower diversity, more fragmented habitat connectivity, or with greater environmental gradients may therefore find alternative results, such as complementarity or additional influence of environmental factors.

### 4.2 Functional traits structuring standing stock biomass

We found support for the dominance hypothesis, specifically that two traits related to fish species’ position in the water column predicted the biomass of a community. This result suggests certain community‐level trait values can enhance EF. Here, both eye position and oral gape position were negatively correlated with community biomass, suggesting that the most productive eelgrass fish communities were those dominated by species such as *Leptocottus armatus,* which are more adapted for a benthic environment. Fish with high values for the eye position metric have more dorsally located eyes and are adapted to capturing prey from the pelagic environment, whereas fish with low scores for eye position have more laterally located eyes and are more adapted for capturing prey from the benthic environment (Gatz, 1979). Similarly, fish with high gape position scores have dorsally located mouths, whereas those with low scores, such as those in the sculpin family (Cottoidea), have ventrally located mouths and are best adapted to capturing prey from the benthic environment (Karpouzi & Stergiou, 2003). Importantly, there was a weaker relationship between body length and community biomass, indicating that it is not simply communities with larger bodied individuals that contain more biomass, but rather these specific ecologically relevant traits. While we focused on traits related to species’ feeding abilities, it is possible that traits related to other life‐history characteristics or environmental tolerances could also influence a species’ dominance within a community (e.g., metabolic rate, parental care habits, pollution tolerance), and this area provides grounds for further research.

The trait values that permit maximum growth and productivity will vary across ecosystems, depending on the distribution of food and habitat resources within them (Keck et al., 2014; McGill et al., 2006). Previous work on fish species in coral, mangrove, and seagrass habitats from the Caribbean and Australia showed that habitat rather than geographic region was most related to functional traits (Hemingson & Bellwood, 2018). The authors reasoned that differences in habitat structure, such as mangrove roots compared to seagrass leaves, their related predation risk, and food availability, outweighed the evolutionary distinctiveness found between ocean regions. In our study system, food availability typically relates to small prey such as amphipods, which they themselves maybe associated with particular habitats (Stoner, 1980). As a result, it may actually be the availability and distribution of prey (Ingram & Shurin, 2009), itself a function of habitat, that determines the optimal traits for accumulation of community biomass. As such, we would expect our findings to be transferable to other habitats with similar biophysical compositions. Specifically, prey in eelgrass systems are known to be benthic and epifaunal (Phillips, 1984), which corresponds with our findings that communities with trait values associated with more benthic prey capture were found to contain the highest community biomass. Adaptations to feed in highly structured benthic habitats are common in seagrass‐associated fish species (Hovel et al., 2016) and may include both morphological and behavioral components. An important future opportunity would be to test if habitats with more similar prey distributions have a strong relationship between community biomass and traits like eye and oral gape position. Such a test would help determine if it is trophic transfer or habitat use that most influences the optimal traits within a community. Centering our understanding of diversity mechanisms on traits allows us to make informative comparisons across systems based on quantifiable features of organisms and their environment.

## 5 MANAGEMENT IMPLICATIONS AND CONCLUSIONS

Our finding that a few dominant species contribute disproportionately to community biomass has important consequences for species and ecosystem management. If we wish to prioritize conserving fish biomass as a function within this ecosystem, we may wish to target those species that contribute the most to this function, in addition to managing habitat to preserve overall biodiversity. This finding highlights the importance of understanding species variation in environmental sensitivities as well as contributions to function. For example, we might choose to prioritize mitigating human disturbances that particularly affect dominant species. Alternatively, since dominant species are less immediately at risk of loss due to stochastic forces, we might conclude that the functional importance of dominant species and the risk of biodiversity loss for rare species each deserve special attention, and monitoring and conservation efforts should be split between these objectives rather than focusing only on rare species. The ethical considerations of these decisions are considerable, and conservationists should be aware of unexpected outcomes when promoting BEF in management and conservation frameworks. It is also salient to consider how we might manage for only one or a few species all sharing the same habitat. If it were more resource‐intensive to only protect these highly functional species, a more prudent approach might be to simply protect the habitat, protect all species, and potentially protect the greatest number of species and functions.

We must also acknowledge the limitations of the methods used, and the functions considered. As an observational study, it is not possible to establish causality but our results provide inferences into BEF links and join a growing body of literature suggesting that dominance may be a key factor to consider (Maureaud et al., 2019). We also used beach seines to survey the fish community during one season, as a result we only accessed a spatial and temporal subset of the possible community; surveys outside of said time and space might yield different results. As multitrophic interactions are taken into consideration, it is also plausible that alternative BEF relationships could be found (see Griffin et al., 2013). Similarly, we know that these species use multiple habitats, typically moving from eelgrass ecosystems to deeper systems as they age (Olson et al., 2019; Phillips, 1984). As nursey habitats (McDevitt‐Irwin et al., 2016), we speculate that most of the biomass recorded in our study was accumulated in eelgrass but may then be transferred to nearby habitats as species move. Studies do not typically consider the effect of BEF across ecosystems (but see van der Plas, 2019), but this consideration could be important for systems such as ours. Lastly, we examined a single function: While one or a few species may drive a particular function, as was found in our study, those same species may not be responsible for other functions in the ecosystem (Isbell et al., 2011). Seagrass ecosystems have well‐established links between fish predators, invertebrate grazers, epiphytic algae, and the eelgrass itself. For example, certain fish predators can prevent herbivorous invertebrates from grazing epiphytic algae off the eelgrass, and the resulting increased epiphyte loads reduce the fitness of the seagrass (Heck & Valentine, 2006; Moksnes et al., 2008). Thus, while fish community assemblages dominated by certain species might maximize community biomass, those same assemblages might result in grazer communities that do not maximize epiphyte control and seagrass growth.

Our results highlight the importance of dominance and relative abundance in driving ecosystem function in eelgrass fish communities, but they also have implications for other ecosystems. First, past research has found support for the dominance hypothesis in producers in forests, pollination services in bees, and secondary productivity in pelagic fisheries (Gilbert et al., 2009; Maureaud et al., 2019; Tardif & Shipley, 2012; Winfree et al., 2015), and our results suggest these relationships span across taxa and could be replicated in other vertebrate communities. Second, many systems have steep rank–abundance curves, where one or a few species dominate community abundance and most species are rare (Ulrich et al., 2010). If these patterns in relative abundance are inherent to the distribution of resources in the system and not a recent feature of system disturbance or invasion, then the contributions of these few species may be central to ecosystem functioning and deserve greater attention (Eisenhauer et al., 2016). Third, communities are more likely to change in relative abundance than to lose richness, at least in the short term (Ceballos et al., 2017). Our results suggest these declines can negatively impact ecosystem function even without complete species loss. As such, we suggest that ecologists place an increased value on studying and maintaining species abundances within a community as opposed to species richness alone.

In this study, we highlight the importance of dominant species, their traits, and their distribution within a community for driving ecosystem function in a real‐world ecosystem. Our work stresses the importance of determining whether we can detect patterns consistent with BEF predictions in multiple natural ecosystems and under different contexts, where different mechanisms may apply. We also stress the importance of examining multiple facets of biodiversity, including abundance weighted measures of diversity, in BEF analyses. Specific to functional diversity, we have also shown the relevance of the currently underused one‐dimensional functional evenness and CWM metrics, and how they can be used to understand which traits and which trait values best explain ecosystem function. Finally, given the importance of dominant species in determining ecosystem function in our system, we conclude that conservation that prioritizes single ecosystem functions may not always maximize diversity, and a greater understanding of how different dimensions of diversity contribute to different dimensions of ecosystem functioning and value is needed.

## CONFLICT OF INTEREST

The authors declare that they have no conflict of interest.

## AUTHOR CONTRIBUTIONS

**Aaron M. Eger:** Conceptualization (equal); Data curation (lead); Formal analysis (lead); Funding acquisition (supporting); Investigation (lead); Methodology (lead); Project administration (lead); Visualization (lead); Writing‐original draft (lead); Writing‐review & editing (equal). **Rebecca J. Best:** Conceptualization (equal); Formal analysis (supporting); Methodology (equal); Supervision (supporting); Writing‐review & editing (equal). **Julia K. Baum:** Conceptualization (equal); Funding acquisition (lead); Methodology (supporting); Project administration (supporting); Resources (lead); Software (lead); Supervision (lead); Validation (equal); Writing‐original draft (supporting); Writing‐review & editing (equal).

## RESEARCH INVOLVING ANIMALS

All applicable international, national, and/or institutional guidelines for the care and use of animals were followed. Fish were collected under a Department of Fisheries of Canada Scientific Fishing Permit (XR 10 2016), a University of Victoria Animal Health and Welfare Permit (2016‐011), a Prince Rupert Port Authority Work Permit, and with consultations with local First Nations, Gitxaala, Git'agaat, Metlakatla, Kitselas, Kitsumkalum and Lax Kw'alaams, on whose traditional territory the work took place.

### OPEN RESEARCH BADGES

This article has earned an Open Data Badge for making publicly available the digitally‐shareable data necessary to reproduce the reported results. The data is available at https://doi.org/10.17605/OSF.IO/6SQRF.

## Supporting information

Appendix S1‐S4

## Data Availability

All the data and the scripts for running the analysis are deposited on Open Science Framework at the link: https://doi.org/10.17605/OSF.IO/6SQRF.
