# Supplementary material for: Dominance determines fish community biomass in a temperate seagrass ecosystem
Source: Ecol Evol. 2021 Jul 6;11(15):10489–501. doi: 10.1002/ece3.7854 (PMC8328455; doi:10.1002/ece3.7854)

**Appendix**

Appendix S1: Correlation values (Pearson’s R) amongst the variables considered.


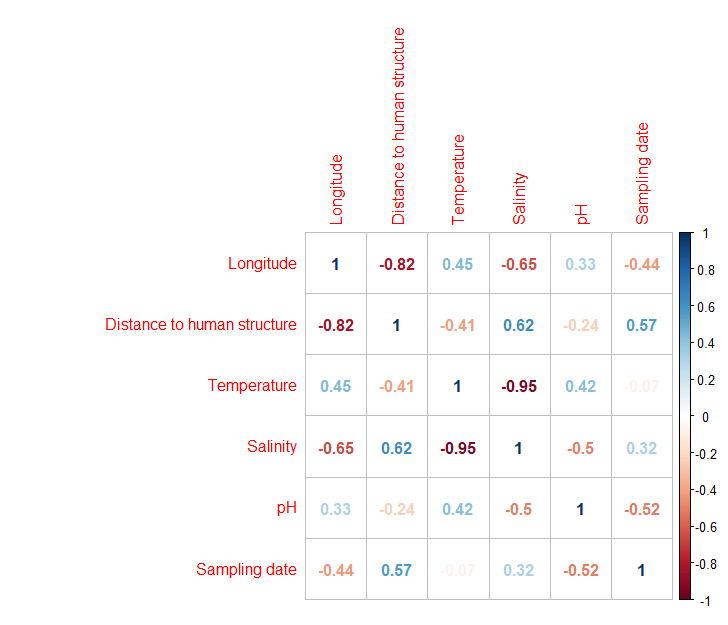


Appendix S2: Species rank abundance plots for the survey sites. Different coloured lines represent the different models used to fit the curves; the fits were done using the radfit function in the “vegan” package in R.


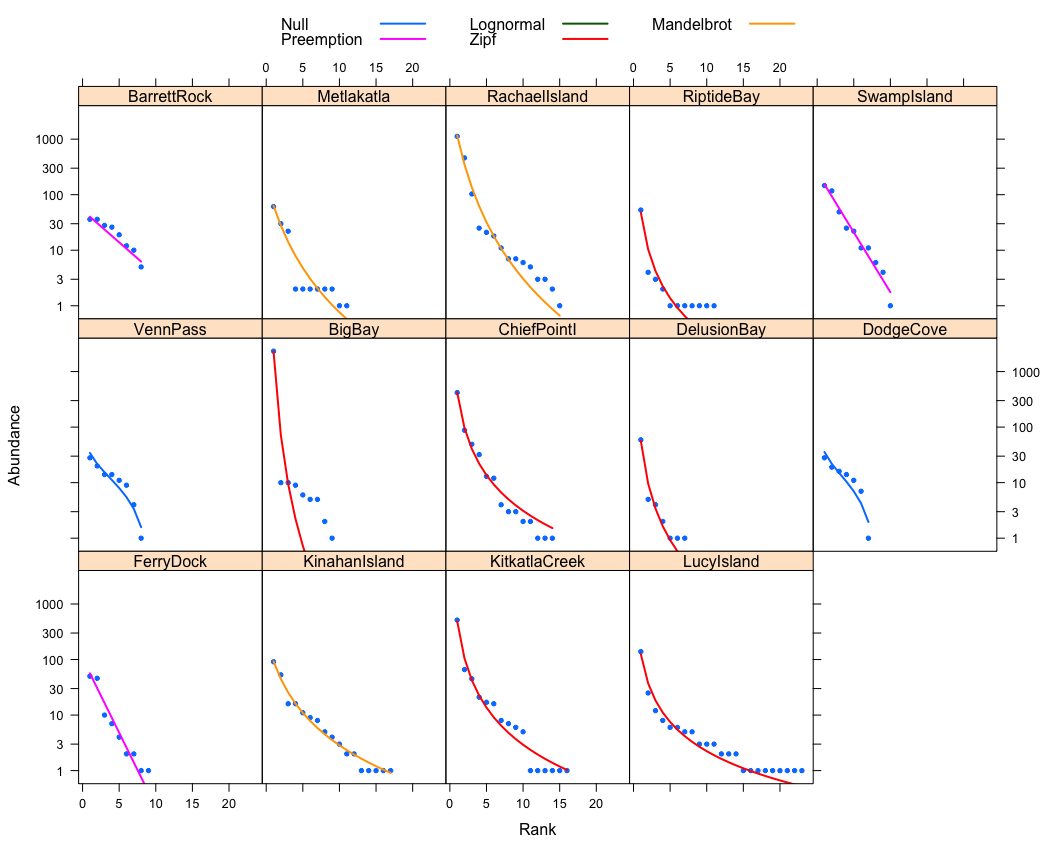


Appendix S3: All species caught and photographed along with the number of sites where species was observed (Regional occupancy, maximum of 14), the median proportion that species contributed to a site’s biomass, and the species’ morphological measurements used to calculate trait values (abbreviations as in. Fig 2). Species are ordered from highest to lowest median proportion of site biomass.

Appendix S4: Relationships between the log site-level fish community biomass and site-level abiotic variables: a) temperature b) salinity c) pH and d) eelgrass density.


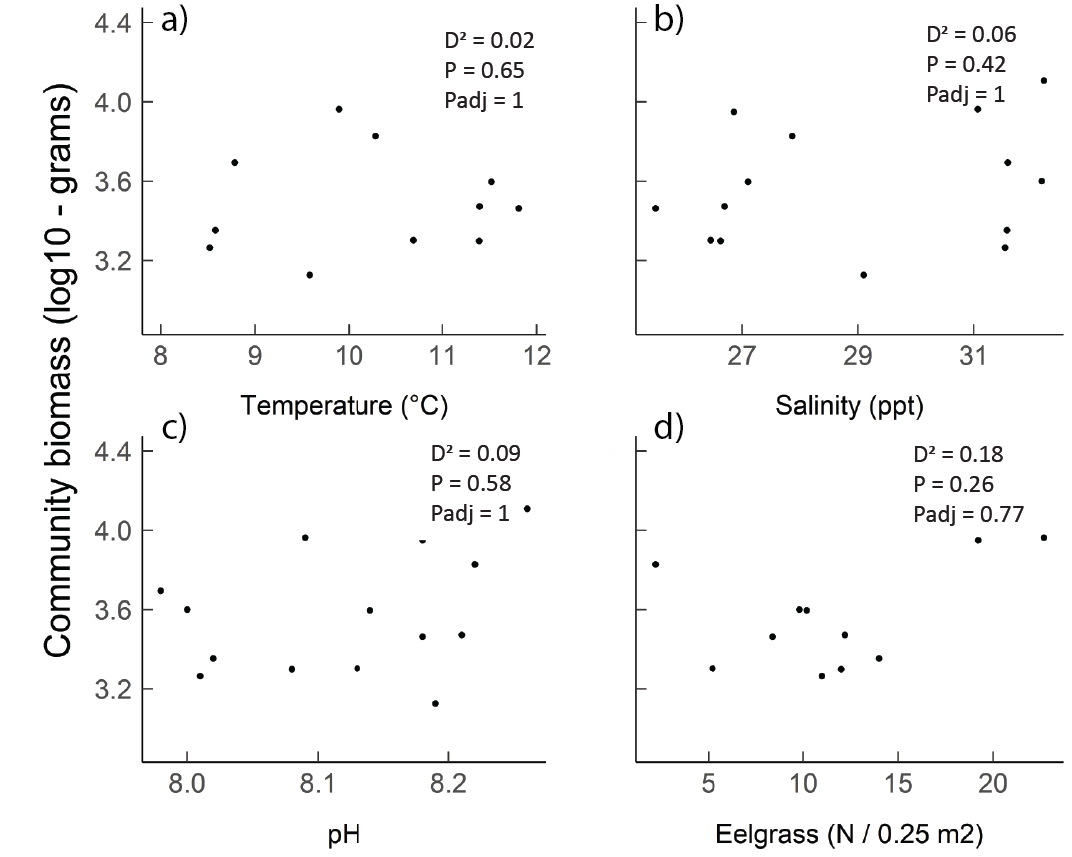

Supplement: Supplementary file 1 — Appendix S1‐S4 [file ECE3-11-10489-s001.docx]
